# A High Throughput Method for Measuring Polycyclic Aromatic Hydrocarbons in Seafood Using QuEChERS Extraction and SBSE

**DOI:** 10.1155/2015/359629

**Published:** 2015-03-19

**Authors:** Edward A. Pfannkoch, John R. Stuff, Jacqueline A. Whitecavage, John M. Blevins, Kathryn A. Seely, Jeffery H. Moran

**Affiliations:** ^1^GERSTEL Inc., 701 Digital Drive, Suite J, Linthicum, MD 21090, USA; ^2^Arkansas Department of Health, Public Health Laboratory, 201 S. Monroe Street, Little Rock, AR 72205, USA; ^3^Department of Pharmacology and Toxicology, University of Arkansas for Medical Sciences, 4301 W. Markham Street, Little Rock, AR 72205, USA

## Abstract

*National Oceanic and Atmospheric Administration (NOAA) Method NMFS-NWFSC-59 2004* is currently used to quantitatively analyze seafood for polycyclic aromatic hydrocarbon (PAH) contamination, especially following events such as the Deepwater Horizon oil rig explosion that released millions of barrels of crude oil into the Gulf of Mexico. This method has limited throughput capacity; hence, alternative methods are necessary to meet analytical demands after such events. Stir bar sorptive extraction (SBSE) is an effective technique to extract trace PAHs in water and the quick, easy, cheap, effective, rugged, and safe (QuEChERS) extraction strategy effectively extracts PAHs from complex food matrices. This study uses SBSE to concentrate PAHs and eliminate matrix interference from QuEChERS extracts of seafood, specifically oysters, fish, and shrimp. This method provides acceptable recovery (65–138%) linear calibrations and is sensitive (LOD = 0.02 ppb, LOQ = 0.06 ppb) while providing higher throughput and maintaining equivalency between NOAA 2004 as determined by analysis of NIST SRM 1974b mussel tissue.

## 1. Introduction

When the Deepwater Horizon oil rig exploded off the northern coast of the Gulf of Mexico on April 20, 2010, millions of barrels of crude oil were released into the gulf before the well was capped, and later sealed, almost six months later. Fragile ecosystems, air and water quality, food supplies, human health, and economies in and around the Gulf of Mexico are still being impacted by this spill [1]. Without effective monitoring of food and water quality after such spills, fisheries could remain closed unnecessarily or products from unmonitored fisheries may enter the general food supply, leading to potential endangerment of public health. Nine PAHs were initially selected as markers for contamination in seafood harvested in and around potentially impacted areas [2]. Regulatory limits and established safe levels of exposure for each of these analytes are summarized in Table 1.

The National Oceanic and Atmospheric Administration is responsible for closing and opening Federal waters for seafood harvest. The NOAA Office of Response and Restoration (OR&R) publication entitled* Managing Seafood Safety after an Oil Spill* [3] and input from NOAA, the Food and Drug Administration, the Environmental Protection Agency, and several state authorities were used to establish criteria for analytically screening seafood for oil contamination as part of the Deepwater Horizon explosion. NOAA currently recommends using* NOAA Method NMFS-NWFSC-59 2004* [4] as the preferred method for quantifying polycyclic aromatic hydrocarbons (PAHs) in seafood harvested from potentially oil-impacted areas. This gas chromatography/mass spectrometry (GC/MS) method recommends running batches of only 12 to 14 samples where assay preparation, sample preparation, and extensive sample cleanup require multiple days of work to complete. Additionally, size-exclusion high-performance liquid chromatography is completed prior to GC/MS analysis for further sample cleanup. Lastly, the GC/MS method is almost an hour long for each sample [4]. All of these steps result in a low-throughput method. Hence, there is concern that the NOAA 2004 method may not have the throughput capacity necessary during an emergency response. Other methods have been utilized to extract PAHs from seafood, like solid-liquid extraction with* n*-hexane or dichloromethane with a mandatory solid phase extraction cleanup step [5], but larger volumes of chlorinated solvents are necessary and relative standard deviations (RSDs) are high for some compounds. Also, solid-phase microextraction (SPME) has been utilized to extract PAHs from seafood, which eliminates the need for solvents and a separate cleanup step [6], but is not high throughput since the SPME fiber is exposed to a single sample for 60 minutes.

As a result of the Deepwater Horizon explosion, a new PAH screening method was developed using liquid chromatography/fluorescence (LC/FL) technology [2]. Although the LC/FL method is fast and high throughput, this method lacks quantification and mass spectral confirmation and is only used for screening purposes [7]. Incorporating existing extraction procedures like QuEChERS (quick, easy, cheap, effective, robust, and safe) may retain the throughput capacity of this method and add much needed quantification capabilities.

QuEChERS was first developed to extract a broad spectrum of pesticides from fruits and vegetables and has been shown to yield high recovery of apolar compounds from a variety of plant materials [8]. The technique has since been extended to other analytes including PAHs in fish tissue with recoveries of >90% [9, 10]. QuEChERS uses a water-miscible solvent to extract analytes of interest but requires dispersive solid phase extraction (dSPE) for further sample cleanup. In dSPE procedures, primary secondary amine (PSA) adsorbent is typically used to remove organic acids while C18 or graphitized carbon black can be included to remove fats and pigments [11–13].

In 1999, Baltussen et al. developed a microextraction technique commonly referred to as stir bar sorptive extraction (SBSE) [14], which initially was used to extract compounds from liquid matrices [15]. This technique uses 1-2 cm magnetic stir bars coated with a 0.5 or 1.0 mm film of polydimethylsiloxane (PDMS), a commonly used sorptive material, to extract compounds with high octanol : water partition coefficients (log⁡⁡*K*_*o*/*w*_ > 2 ) [14, 16]. SBSE is easy to use and parts per billion detection levels for apolar pollutants, like PAHS, in aqueous solutions are possible when combined with GC/MS [17, 18]. Also, SBSE has been successfully applied for the detection and quantification of trace levels of numerous analytes in food, environmental, and forensic applications [15, 19, 20]. Further, the United States Environmental Protection Agency Region 7 Laboratory concluded that SBSE can meet* EPA Method 625* performance criteria for all 18 PAHs listed [21].

The purpose of this study is to determine if QuEChERS and SBSE technology can be combined to provide an extraction and concentration procedure for PAHs from fish and shellfish that can be coupled to GC/MS. This new extraction method will result in a high throughput approach, unlike the NOAA 2004 method, with quantifiable results and mass spectral confirmation, unlike the LC/FL method. This method is the first to combine QuEChERS with SBSE technology to successfully develop a method to minimize matrix interference and significantly increase sample throughput while maintaining quantifiable results for low level measurements.

## 2. Materials and Methods

### 2.1. Reagents and Chemicals

Analytical PAH standards (part number 31458) and deuterated Semi-Volatile Internal Standard Mix (part number 31006) were from Restek (Bellefonte, PA, USA), while optima LC-MS grade acetonitrile (ACN), methanol, dichloromethane, and sodium hydrogen carbonate were all purchased from Sigma Aldrich (St. Louis, Missouri, USA). Deionized (DI) water used for this work was purified to 18.2 M*Ω*-cm resistivity. NIST (Gaithersburg, MD, USA) standard reference material was organics in mussel tissue (SRM 1974b). QuEChERS AOAC extraction kits containing 6.0 g MgSO_4_ and 1.5 g sodium acetate were provided as generous gifts from Agilent Technologies (Santa Clara, CA, USA). Stir bars were conditioned using a TC-2 tube conditioner (GERSTEL, Linthicum, MD, USA). Unless otherwise specified all other chemicals and reagents were of reagent grade or higher.

### 2.2. Equipment

Studies were performed in two different laboratories with slightly different instrumentation as follows: Sample tissue was homogenized using (1) equal parts sample and DI water using a Waring (Lancaster, PA, USA) model LB10S variable speed steel bowl lab blender, or (2) homogenized frozen with a Robot Coupe (Jackson, MS, USA) RSI 2Y1 laboratory grade blender by incorporating a small amount of dry ice. Samples were agitated either manually or with an ATR (Laurel, MD, USA) RKVSD Rotamix rotating inverter. All extracts were centrifuged with either a Thermo Fisher Scientific (Waltham, MA, USA) Sorvall Evolution RC centrifuge or Eppendorf (Hamburg, Germany) 5430R centrifuge. SBSE was performed at room temperature with GERSTEL Twister stir bars in 10 mL headspace vials on a 20 position magnetic stir plate. Stir bars were thermally desorbed using a TDU thermal desorption unit with a CIS 4 programmed temperature vaporizing inlet and analysis automated using an MPS 2 autosampler with Maestro software (GERSTEL, Linthicum, MD, USA). Lastly, an Agilent (Santa Clara, CA, USA) 7890 GC interfaced with either an Agilent 5975 or 5973 MS was used.

### 2.3. Standard and Sample Preparation

Samples of frozen gulf shrimp, fresh oysters, and Atlantic croaker (*Micropogonias undulatus*) were either obtained from local markets or as a generous gift from the Alabama Public Health Laboratory. Seafood obtained at local markets was purchased whole to verify identification prior to being homogenized. Sample tissue (fresh or partially frozen) was homogenized either with equal parts water or by using dry ice in a laboratory grade blender. No difference in results was seen between either of preparation methods. Dry ice was sublimed from homogenized samples at −20°C prior to analysis. Homogenates equivalent to 3.0 ± 0.1 g tissue (or 6.0 ± 0.1 g of water homogenate) were weighed into 50 mL conical tubes. Internal standard and PAH standard solutions were spiked directly onto the tissue in the tube.

Stock solutions of PAH standards, quality controls, and internal standards were prepared by diluting original solutions in ACN to yield 0.3, 3.0, 15, 30, and 75 ng/*μ*L spiking solutions. Stock solutions of deuterated PAH standards used as internal standards were prepared by diluting original solutions in ACN to yield a spiking solution of 3.75 ng/*μ*L. Adding 20 *μ*L of internal standard to 3.0 g tissue (or 6.0 g of water homogenized tissue) yielded an internal standard concentration of 25 ng/g in tissue. Adding 10 *μ*L of each standard spike level to 3.0 g tissue (or 6.0 g water homogenized tissue) yielded spike levels of 1.0, 10, 50, 100, and 250 ng/g.

We performed the standard AOAC version of QuEChERS without further optimization by following package insert directions. In brief, DI water was added to samples to normalize final weight at 15 g. Samples were vortexed for 30 seconds and further diluted with 15 mL of ACN and vortexed for an additional minute. The contents of the QuEChERS salt packet (6.0 g MgSO_4_ and 1.5 g sodium acetate) were added to the sample and shaken 1 minute. Samples were mixed on an ATR rotator for 10 minutes and centrifuged at approximately 4000 ×g for 5 minutes at 5.0°C. The upper ACN layer was collected and stored up to 48 hours prior to analysis.

Prior to use, Twister stir bars were conditioned at 300°C under 80 mL/min zero grade nitrogen flow in the tube conditioner for 2 hours. SBSE was accomplished by transferring 1.0 mL aliquots of the upper ACN layer to a 10 mL headspace vial containing a conditioned, precoated stir bar and 4.0 mL of 0.1 M NaHCO_3_ to reduce organic acid interference. Samples were stirred at room temperature for 90 minutes at approximately 1200 rpm. Stir bars were removed with clean tweezers, rinsed briefly with DI water, blotted dry, and placed into clean glass desorption tubes for analysis.

Calibration of the thermal desorption unit was performed by spiking a known amount of PAH standard mix onto Tenax TA adsorbent tubes (Supelco, Bellefonte PA, USA) and desorbing under the same conditions as used for the Twister desorption, as described below. The total recovery of the combined QuEChERS/SBSE procedure was determined by using this calibration to quantify the PAHs recovered from the spiked oyster matrix.

### 2.4. Stir Bar Desorption and GC/MS Conditions

Stir bars were thermally desorbed at 100 mL/minute into the GC using the thermal desorption unit in splitless mode heated at 720°C/minute from 40°C (0.2 min) to 300°C (5 minutes). Analytes were refocused in the inlet at −120°C on a quartz wool-filled liner in solvent venting mode and transferred to the column by heating the inlet at 720°C/minute to 300°C (3 minutes) with a 10 : 1 split ratio. Since the focus of this study was the extraction and cleanup procedure, adequate chromatographic separation was performed on a Restek DB 5MS or 5XI 5SIL MS column (30 m × 0.25 mm × 0.25 *μ*m) with zero grade helium carrier at 1 mL/minute constant flow unless otherwise noted. The column was held at 60°C for 1 minute and then heated at 15°C/min to 325°C and held for 3 minutes for a total run time of 21.7 minutes. Simultaneous detection was performed using selective ion mode (SIM)/Scan mode from 50–400 amu. Specific SIM parameters including the quantifier and two qualifier ions are provided in Table 2.

### 2.5. GERSTEL Twister Stir Bar Cleaning Procedures

After use, stir bars were cleaned by soaking 10–40 stir bars overnight in 40 mL of a 50% methylene chloride in 50% methanol solution. The liquid was poured off and stir bars were carefully spread out on a clean watch glass in the fume hood to allow excess solvent to evaporate for 2 hours. Twisters were thermally conditioned at 300°C in the tube conditioner under a stream of nitrogen (80 mL/min per tube) for 2 hours and allowed to cool for 15 minutes under nitrogen flow before being stored individually in 2 mL vials. Stir bars analyzed after cleaning showed no detectable PAH carryover (data not shown).

## 3. Results and Discussion

Combining QuEChERS and SBSE technology increases sample throughput with quantitative results. Data show that the combination of QuEChERS and SBSE is a viable approach for the determination of important PAH markers in oysters and other seafood that eliminates extensive sample preparation and increases quantitative throughput capabilities.

Although the QuEChERS technique has been used previously to extract PAHs in fish [10, 22], the procedure does not provide additional concentration unless the final sample is evaporated and reconstituted, which also simultaneously concentrates any remaining matrix interferences. Furthermore, using a dSPE cleanup step appears to leave some amount of matrix compounds in the sample extract that may interfere with the analysis [22, 23]. Large volume injection has been used to improve the detection limits for pesticides after QuEChERS extraction [24, 25], but retention time shifts were reported after only three injections on the GC/MS unless back-flushing was used to eliminate interference from high molecular weight matrix contaminants [23]. These initial attempts at using only QuEChERS failed to provide adequate detection limits; therefore, we incorporated SBSE as a dual cleanup and concentrating step.

### 3.1. Optimizing SBSE Conditions

**Figure 1:**
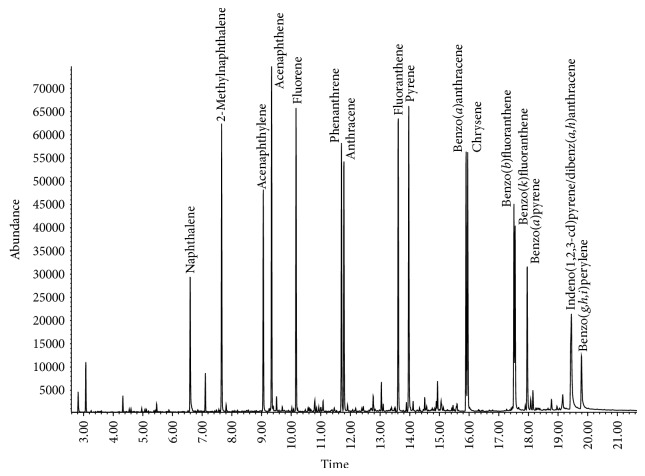
Total ion chromatography of PAHs in oysters spiked at 25 ng/g.

**Figure 2:**
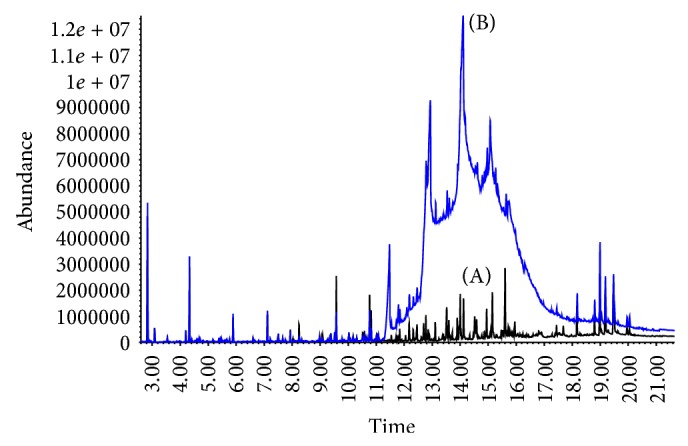
Matrix interference is reduced in oysters during SBSE with use of 0.1 M NaHCO_3_ (A) instead of water (B).

Since PAHs are ubiquitous, precautions must be taken to ensure background contamination is minimized. Oysters were chosen as the first matrix to test the QuEChERS/SBSE method in seafood because oysters are considered a difficult matrix since the high fat content in oysters may introduce high background interferences [12]. Prior to this study, we have performed SBSE in aqueous solutions containing water-miscible organic solvents for compounds with high *K*_*o*/*w*_ and found the relative percentage of organic solvent must be optimized for efficient extraction of the target analytes. Based on our previous studies and a study by Ochiai et al. [16], 20% ACN was selected because the log⁡⁡*K*_*o*/*w*_ for the PAHs of interest ranged between 3.3 and 6.75. Therefore, to perform the SBSE on the QuEChERS extracts, 1.0 mL of the acetonitrile layer was diluted into 4.0 mL water or buffer resulting in a final solution containing 20% ACN. When the optimized extraction conditions were used with the oyster matrix, including the addition of 0.1 M NaHCO_3_, excellent signal to noise ratio in the SIM mode was obtained (Figure 1). SBSE also provided a concentration factor up to 1000x compared to liquid injection, which enabled quantification of very low levels of analytes. Preliminary SBSE extraction studies evaluated extraction times of 30, 60, and 90 minutes, 4 hours, and 16 hours (overnight) to estimate near-equilibrium conditions. Based on these studies, SBSE extraction time for this matrix was evaluated at 90 minutes or 16 hours. No significant improvement in signal was seen with overnight extraction (data not shown); therefore, a 90-minute extraction was used for all subsequent testing. In addition, incorporating 0.1 M NaHCO_3_ during SBSE greatly reduced interference due to organic acids and improved signal-to-noise ratios (Figure 2 (A and B)). With routine instrument maintenance, the optimized conditions provided stable chromatography for >200 samples.

### 3.2. Method Linearity and Recovery

GC/MS analysis was used to determine extraction performance, but optimization of the GC/MS parameters was secondary to investigating the combination of QuEChERS and SBSE as a novel extraction procedure for PAHs in seafood. The Agilent 5975 GC/MS configuration used in this study was ideal due to the ease of use to evaluate extraction performance and general availability and robust high throughput nature of the instrumentation. The described extraction procedure can be used to introduce sample into any optimized GC/MS configuration where the columns, GC/MS conditions, or other instrumentation, like GC/QQQ, could be used to analyze these extracts. Normalizing instrument responses to deuterated PAH internal standards produced linear calibrations and accounted for varying extraction efficiencies (Table 3). The QuEChERS/SBSE method provides linear calibration with concentrations of 1, 10, 50, 100, and 250 ng/g matrix for 9 target PAHs (Table 3) with recoveries of 65.5–138.4% for concentrations spiked at 2.5, 50, and 250 ng/g in oysters (Table 3, *n* = 3 for each spiked concentration), which is consistent with previous studies using this technology [10, 17, 18].

### 3.3. Method Trueness and Precision

Trueness and precision of these determinations were further assessed by measuring PAH concentrations in oysters spiked at 2.5, 50, and 250 ng/g (Table 3). Analysis of a NIST standard reference material performed over a period of several weeks illustrates the accuracy and precision of the method using certified reference material (Table 4). This analysis provided excellent recovery for total PAH (98%, *n* = 11) with high precision (7–19% RSD) that meets the guidelines expressed in the NOAA reference method and National Institute of Standards and Technology Deepwater Horizon study [1, 26]. Only two PAH values, naphthalene and anthracene, were outside the target range. Naphthalene exhibited the highest variability (19% RSD) which may have been due to losses during the extra 10-minute shake in our QuEChERS procedure and the relatively high volatility of naphthalene. Although the value for fluorene is within range of the NIST standard reference material, the fluorene values reported in Table 4 were only detected using a 60 m column (*n* = 3) because of coelution of other analytes, such as PCBs, that was difficult to separate using the shorter 30 m column. Anthracene was overestimated due to coelution of contaminants when a shorter GC separation was employed for the study. The presence of a coeluting contaminant in the stir bar extract was confirmed by comprehensive GC × GC analysis on a Leco Pegasus 4D system (data not shown). All of the PAHs were detected, even anthracene, much below the lowest regulatory limits. The limits of detection for the PAHs were found to be 0.020 ppb and the limits of quantification 0.060 ppb, based on* s*/*n* ratios of 3 : 1 and 10 : 1, respectively.

### 3.4. Testing Additional Matrices

Since the SBSE method was successfully validated in both spiked oysters and in NIST standard reference material (mussel tissue) the method was repeated in other seafood matrices using croaker (a finfish) and shrimp.  Table 5 shows the linear regression data with 1–250 ng/g spiked tissues with mean *r* ^2^ values from 0.9905–0.9948 (*n* = 3). Also, percent recoveries ranged from 63.1 to 93.6% when croaker and shrimp were spiked with 50 ng/g of analyte, a concentration below the regulatory level of concern. Even though further inter- and intralaboratory studies are needed to fully test the utility of this new extraction procedure, these results with oysters, croaker, and shrimp show significant promise for increasing analytical capacity for assessing petroleum contamination in potentially impacted seafood.

The SBSE method is successful with difficult matrices, like seafood, by not only decreasing matrix interference, but also concentrating the analytes. The method and theory of SBSE has been described in detail elsewhere [27]. Briefly, the PDMS coating on the stir bar acts as an immobilized liquid into which apolar analytes in an aqueous matrix can partition. Because of the apolar nature of the PDMS, polar matrix components (including inorganic salts, carbohydrates, ionized acids, and amines) do not partition well into the PDMS and therefore do not interfere with the analysis. Furthermore, since loading capacity is based on the volume of PDMS on the stir bar, not the surface area, high molecular weight apolar components such as triglycerides and peptide fragments, which do not diffuse effectively into the PDMS layer, will not interfere with the analysis. Hence, SBSE provides sample cleanup as well as sample concentration and achieves low detection limits in complex sample matrices.

### 3.5. Method Workflow and Throughput

**Figure 3:**
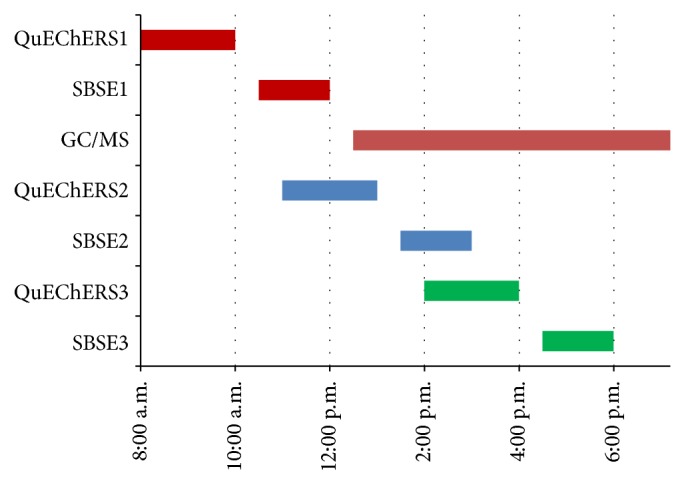
Flowchart demonstrating high throughput application of QuEChERS/SBSE method with one technician completing about 60 samples per workday.

The QuEChERS/SBSE method is inexpensive since the stir bars can be cleaned and reused for 30–50 samples, resulting in an estimated cost of <$10 per sample for all consumables, including QuEChERS kits. In addition, we propose that the QuEChERS/SBSE procedure can provide high sample throughput. The workflow possible by a single analyst is illustrated in Figure 3. The red bars represent the sample processing steps for a single batch of 20 samples prepared by first performing QuEChERS extractions on a batch of 20 samples followed by unattended SBSE for 90 minutes. Samples are loaded onto the GC/MS and an automated analysis is started. It is possible for a single analyst to prepare a second batch of 20 samples (blue bars) and even a third 20-sample batch (green bars) per day which can be added to the automated GC/MS analysis. The length of the GC/MS method, or other analysis, may vary, but results can be obtained for about 60 samples per day.

## 4. Conclusion

The QuEChERS/SBSE method presented is a viable alternative to the NOAA method and the LC/FL method to maintain sensitivity, accuracy, and precision while efficiently quantifying PAH contamination in seafood. The QuEChERS extraction strategy was used to effectively extract PAHs from complex seafood matrices including mollusks, crustaceans, and finfish. This study uses a novel SBSE with a buffered diluent to concentrate PAHs and eliminate matrix interference from QuEChERS extracts of seafood, specifically oysters, fish, shrimp, and mussels. This method provides acceptable recovery (65–138%) and linear calibrations and is sensitive (LOD = 0.02 ppb, LOQ = 0.06 ppb) while providing higher throughput and maintaining equivalency between NOAA 2004 as determined by analysis of NIST SRM 1974b mussel tissue.

## Figures and Tables

**Table 1:** United States PAH regulatory limits for reopening impacted areas.

Chemical^1^	Levels of concern (ppm)			
	mg/kg/day	13 g/day (shrimp and crabs)	12 g/day (oysters)	49 g/day (finfish)
Naphthalene	0.02	123	133	32.7
Fluorene	0.04	246	267	65.3
Anthracene/phenanthrene	0.3	1846	2000	490
Fluoranthene	0.3	246	267	65.3
Pyrene	0.03	185	200	49
Benz[*a*]anthracene	0.0002	1.32	1.43	0.35
Chrysene	0.02	132	143	35
Benzo[*a*]pyrene	0.00002	0.132	0.143	0.035

^1^Includes alkylated homologues, specifically C-1, C-2, C-3, C-4 naphthalenes; C-1, C-2, C-3 fluorenes; C-1, C-2, C-3 anthracenes/phenanthrenes; C-1, C-2 pyrenes.

Table modified from FDA 2010 [2].

**Table 2:** MSD SIM method conditions; quantifier in bold.

Group	RT^1^ (min)	Ions monitored
1	2.5	(102, 50), (126, 50), (**128**, 50)
2	7.6	(115, 50), (141, 50), (**142**, 50)
3	9.0	(151, 20), (**152**, 20), (153, 20), (154, 20)
4	10.0	(165, 50), (**166,** 50), (167, 50)
5	11.5	(176, 50), (**178**, 50), (179, 50)
6	13.5	(200, 50), (**202**, 50), (203, 50)
7	15.5	(226, 50), (**228**, 50), (229, 50)
8	17.4	(126, 50), **(252**, 50), (253, 50)
9	19.2	(138, 20), (139, 20), (**276**, 20), (277, 20), (278, 20), (279, 20)

Group 1: naphthalene; Group 2: 2-methylnaphthalene; Group 3: acenaphthylene, acenaphthene; Group 4: fluorene; Group 5: phenanthrene, anthracene; Group 6: fluoranthene, pyrene; Group 7: benzo[*a*]anthracene, chrysene; Group 8: benzo[*b*]fluoranthene, benzo[*k*]fluoranthene, Benzo[*a*]pyrene; Group 9: benzo[*ghi*]perylene.

^1^RT is the retention time for the start of the SIM group window.

**Table 3:** Retention time, linear regression, and recovery in oysters.

	RT^1^ (min)	Mean *r* ^2^	Percent recovery (mean ± SEM^2^)		
			2.5 ng/g	50 ng/g	250 ng/g
Naphthalene	5.5	0.9916	125.6 ± 0.08	71.6 ± 0.03	82.4 ± 0.02
Fluorene	9.25	0.9912	92.8 ± 0.02	75.3 ± 0.3	84.5 ± 0.03
Phenanthrene	10.93	0.9957	118 ± 0.07	69.8 ± 0.02	81.5 ± 0.02
Anthracene	11.02	0.9960	95.6 ± 0.03	66.8 ± 0.01	79.9 ± 0.02
Fluoranthene	13.02	0.9937	138.4 ± 0.02	88.7 ± 0.03	100.9 ± 0.03
Pyrene	13.42	0.9937	131.1 ± 0.03	86.1 ± 0.03	97.6 ± 0.03
Benz[*a*]anthracene	15.52	0.9930	90.7 ± 0.02	69.4 ± 0.01	84.3 ± 0.02
Chrysene	15.58	0.9934	103.7 ± 0.03	66.3 ± 0.01	80.7 ± 0.02
Benzo[*a*]pyrene	17.86	0.9940	70.8 ± 0.09	65.5 ± 0.01	81.1 ± 0.03

^1^RT is the retention time.

^2^SEM is the standard error of the mean.

**Table 4:** Analysis on SRM mussel tissue.

Analyte	Acceptable range (ng/g)	Certificate of analysis (ng/g)	QuEChERS-SBSE (ng/g)	%RSD
Naphthalene	1.6–3.3	2.4	1.0	18.5
Fluorene	0.3–0.7	0.49	0.35^1^	14.7
Phenanthrene	1.7–3.5	2.6	1.9	11.1
Anthracene	0.3–0.8	0.53	2.4^2^	15.2
Fluoranthene	11.5–23.1	17	19.2	7.0
Pyrene	12.2–24.2	18	19.0	7.2
Benz[*a*]anthracene	2.9–6.9	4.7	3.7	7.2
Chrysene + Triphenylene	7.4–13.8	10.6	8.8	6.8
Benzo[*a*]pyrene	2.0–3.6	2.8	1.7	10.4

Total		**59.12**	**58.05**	

^1^ *n* = 3 (only detected using 60 m column).

^2^Possible coelution.

**Table 5:** Analysis of finfish and shrimp matrices, *n* = 3.

Spike level (*η*g/g)	Croaker	Shrimp	Croaker	Shrimp
	50	50		
	Percent recovery		Mean *r* ^2^	
Naphthalene	70.5 ± 0.001	70.5 ± 0.017	0.9943	0.9905
Fluorene	63.1 ± 0.003	78.6 ± 0.024	0.9912	0.9930
Phenanthrene	70.7 ± 0.007	67.3 ± 0.004	0.9948	0.9932
Anthracene	69.1 ± 0.008	67.7 ± 0.003	0.9951	0.9810
Fluoranthene	93.6 ± 0.024	78.2 ± 0.008	0.9919	0.9940
Pyrene	85.7 ± 0.024	76.3 ± 0.009	0.9918	0.9920
Benz[*a*]anthracene	64.4 ± 0.016	68.7 ± 0.008	0.9908	0.9929
Chrysene	65.3 ± 0.023	66.9 ± 0.011	0.9916	0.9933
Benzo[*a*]pyrene	64.1 ± 0.006	65.2 ± 0.007	0.9919	0.9915
